# Encapsulated di­chloro­ethane-mediated inter­locked supra­molecular polymeric assembly of A1/A2-dihydroxy-oct­yloxy pillar[5]arene 1,2-di­chloro­ethane monosolvate

**DOI:** 10.1107/S2056989018013415

**Published:** 2018-09-25

**Authors:** Talal F. Al-Azemi, Mickey Vinodh, Abdirahman A. Mohamod, Fatemeh H. Alipour

**Affiliations:** a Department of Chemistry, Kuwait University, PO Box 5969, Safat 13060, Kuwait

**Keywords:** oct­yloxy pillararene, hy­droxy functionalization, crystal structure

## Abstract

Crystals of 1-(1,4-dihy­droxy)-2,3,4,5-(1,4-dioct­yloxy)-pillar[5]arene, C_99_H_158_O_10_·C_2_H_4_Cl_2_, were grown from a 1,2-dicholoro­ethane/*n*-hexane solvent system. In the crystal, the encapsulated 1,2-di­chloro­ethane solvent is stabilized by C—H⋯π inter­actions and mediates the formation of an inter­locked supra­molecular polymer *via* C—H⋯Cl inter­actions.

## Abstract 2

The structural and supra­molecular features of a dihy­droxy-functionalized oct­yloxy-pillararene are discussed. In the crystal, the encapsulated 1,2-di­chloro­ethane solvent is stabilized by C—H⋯π inter­actions and mediates the formation of an inter­locked supra­molecular polymer *via* C—H⋯Cl inter­actions.

## Chemical context   

Supra­molecular polymers constructed by reversible non-covalent inter­actions such as hydrogen bonds, metal–ligand inter­actions, host–guest inter­actions, π–π inter­actions and van der Waals forces have gained considerable inter­est for their intriguing properties of recycling and responsiveness to external stimuli (Raghupathi *et al.*, 2014 ▸; Takashima *et al.*, 2017 ▸). Pillararenes are unique three-dimensional macrocyclic compounds which possess symmetric rigid structures and are easy to functionalize with various substituents (Ogoshi *et al.*, 2008 ▸; Al-Azemi *et al.*, 2017 ▸). They exhibit outstanding abilities to selectively bind different kinds of guest mol­ecules and thus are excellent host mol­ecules for guest encapsulation and mol­ecular recognition. Their unique structural features also enable them to exhibit inter­esting self-assembling characteristics, which make them potential candidates for use in fabricating functional materials in supra­molecular systems and nanotechnology. The construction of pillararene-based supra­molecular assemblies is very inter­esting because it raises the possibility of using these macrocycles for many important functional materials, which include enzyme models, field-effect transistors, gas sensors or photovoltaic cells (Han *et al.*, 2015 ▸; Pan & Xue, 2013 ▸; Hu *et al.*, 2016 ▸; Zhang *et al.*, 2018 ▸).

Supra­molecular motifs such as hydrogen bonding or host–guest inter­actions can be employed to promote the self-assembly of pillararene analogues. The introduction of appropriate peripheral functionalization at the macrocycle will give rise to numerous features that also allow their organization at a supra­molecular level (Xue *et al.*, 2012 ▸). The characteristics of the encapsulated guest mol­ecules can also be utilized to tune the supra­molecular nature of these macromolecules. The present work discusses the crystal structure of a pillararene system, **Pil-OctOH·C_2_H_4_Cl_2_**, which possesses two hy­droxy groups at the macrocyclic periphery. The remaining apical sites on the pillararene are functionalized with long *n*-oct­yloxy substituents. The role of the guest mol­ecule in the formation of an inter­locked supra­molecular polymer *via* various supra­molecular inter­actions is also described.
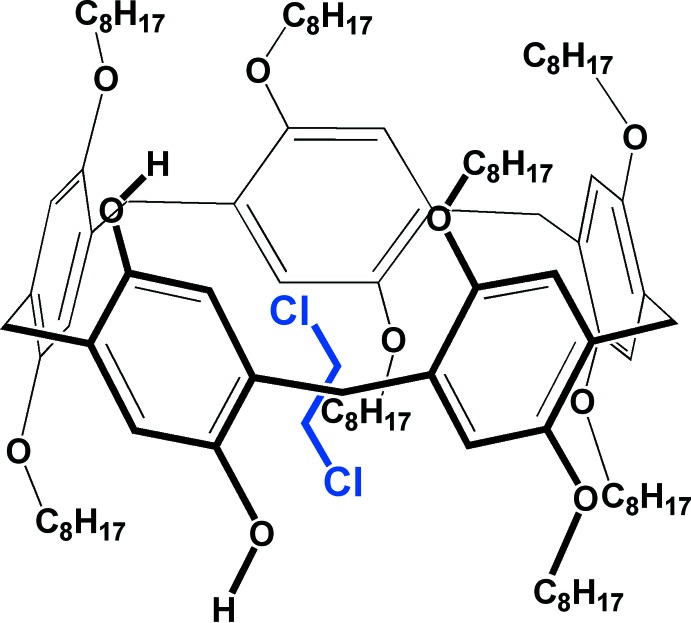


## Structural commentary   

**Figure 1:**
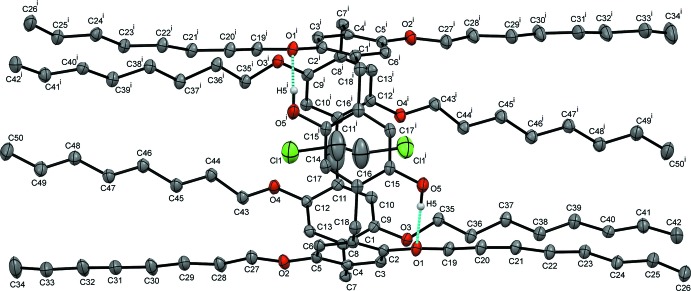
Displacement ellipsoid representation (30% probability) of **Pil-OctOH·C_2_H_4_Cl_2_**. Hydrogen atoms are omitted for clarity except for those of the hy­droxy groups. Blue dotted lines indicate intra­molecular hydrogen bonds between the hy­droxy groups and the oxygen atoms of adjacent oct­yloxy moieties. [Symmetry code: (i) −*x* + 1, *y*, −*z* + 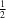.]

Fig. 1 ▸ shows the structure of the title A1/A2-dihy­droxy-oct­yloxy-pillar[5]arene (**Pil-OctOH**). The asymmetric unit contains half of the mol­ecule and the whole structure is generated by twofold rotation symmetry (symmetry operation: −*x* + 1, *y*, −*z* + 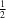). The 1,2 di­chloro­ethane solvent is encapsulated within the pillararene cavity. The basic pillar[5]arene macrocycle is a penta­gon with an average corner-to-centroid distance of 4.99 Å. As a result of the presence of eight linear *n*-oct­yloxy chains at its apical positions, this novel pillararene could be considered to be a long cylindrical-shaped functional mol­ecule where the long tail ends are hydro­phobic in nature. Additionally, the presence of hy­droxy groups at two apical positions provides a hydro­philic pocket in the vicinity of the pillararene macrocycle. The hydroxyl groups are observed to be engaged in intra­molecular hydrogen bonds with the oxygen atoms of the adjacent oct­yl­oxy moieties *via* O—H⋯O inter­actions (Fig. 1 ▸ and Table 1 ▸).

## Supra­molecular features   

**Figure 2:**
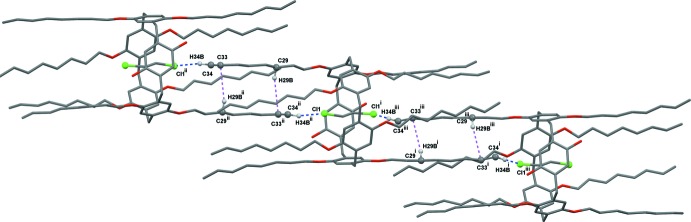
Supra­molecular propagation of **Pil-OctOH** moieties as one-dimensional chains mediated by di­chloro­ethane mol­ecules *via* C—H⋯Cl and C—H⋯ C inter­actions. C—H⋯Cl inter­actions are represented in blue and C—H⋯C inter­actions in purple. [Symmetry codes: (i) −*x* + 1, *y*, −*z* + 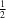; (ii) −*x* + 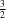, −*y* + 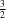, −*z*; (iii) *x* − 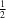, −*y* + 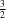, *z* + 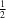.]

**Figure 3:**
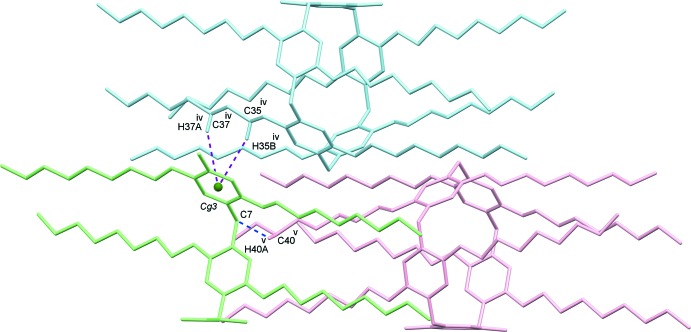
Adjacent pillararene fragments are connected by weak C—H⋯C and C—H⋯π inter­actions in the crystal. Those inter­actions that are involved in supra­molecular pillararene chain formation are omitted for clarity. *Cg*3 is the centroid of the C8–C13 ring. [Symmetry codes: (iv) −*x* + 1, −*y* + 2, −*z* + 1; (v) −*x* + 1, *y*, −*z* + 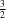.]

In the title macrocyclic compound, the encapsulated 1,2-di­chloro­ethane solvent is stabilized inside the cavity by C—H⋯π inter­actions with the pillararene aromatic ring (Table 2 ▸). Inter­estingly, the guest 1,2-di­chloro­ethane facilitates the formation of a supra­molecular inter­locked network through efficient C—H⋯Cl inter­actions (Fig. 2 ▸ and Table 1 ▸), which form chains along the *b*-axis direction. Additional stabilization of these chains is attained by dimer formation *via* weak C—H⋯C inter­actions between pillararene octyl chains (Fig. 2 ▸ and Table 2 ▸). Although the A1/A2 dihy­droxy groups on the pillararene rim play no part in the formation of the supra­molecular assembly, their small size provides an opening which enables access to the encapsulated guest mol­ecule. The pillararene mol­ecule in each chain inter­acts with neighboring pillararenes of adjacent chains by C—H⋯C and C—H⋯π inter­actions, as given in Fig. 3 ▸ and Table 2 ▸.

## Synthesis and crystallization   

The synthesis of 1-(1,4-dihy­droxy)-2,3,4,5 (1,4-dioct­yloxy)-pillar[5]arene (**Pil-OctOH**) has been reported earlier (Al-Azemi *et al.*, 2018 ▸). Good quality single crystals of this compound were obtained by dissolving the pillararene (25 mg) in 1,2-di­chloro­ethane (0.5 mL) in a small vial and allowing solvent diffusion by keeping this solution in a larger vial containing *n*-hexane (5 ml). Within three days, crystals of the title compound of a suitable size for diffraction analysis had formed.

## Refinement   

Crystal data, data collection and structure refinement details are summarized in Table 3 ▸. The OH hydrogen atoms were located in the electron density map. All other hydrogen atoms were placed at calculated positions and refined using a riding model with C—H = 0.95–0.99 Å and *U*_iso_(H) = 1.2 or 1.5*U*_eq_(C).

## Supplementary Material

Crystal structure: contains datablock(s) I. DOI: 10.1107/S2056989018013415/dx2009sup1.cif

Supporting information file. DOI: 10.1107/S2056989018013415/dx2009Isup4.mol

Structure factors: contains datablock(s) I. DOI: 10.1107/S2056989018013415/dx2009Isup5.hkl

CCDC reference: 1868738

Additional supporting information:  crystallographic information; 3D view; checkCIF report

## Figures and Tables

**Table 1:** Hydrogen-bond geometry (Å, °)

*D*—H⋯*A*	*D*—H	H⋯*A*	*D*⋯*A*	*D*—H⋯*A*
O5—H5⋯O1	0.85 (2)	1.93 (2)	2.754 (2)	165 (2)
C34^i^—H34*B* ^i^⋯Cl1	0.98	2.90	3.782 (3)	151

Symmetry code: (i) 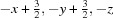.

**Table 2:** Summary of weak inter­actions (Å, °) *Cg*1–*Cg*4 are the centroids of the C15–C17/C15^i^–C17^i^, C1–C6, C8–C13 and C1^i^–C6^i^ rings, respectively.

*D*—H⋯*A*	H⋯*A*	*D*⋯*A*	*D*—H⋯*A*
C51*A*—H51*B*⋯*Cg1*	2.77	3.700 (8)	156
C51*A*—H51*A*⋯*Cg2*	3.04	3.850 (9)	140
C51*B*—H51*D*⋯*Cg3*	2.71	3.565 (7)	144
C51*B*—H51*C*⋯*Cg4*	3.11	4.086 (7)	169
C29^ii^—H29*B* ^ii^⋯C33	3.18	4.136 (3)	163
C35^iv^—H35*B* ^iv^⋯*Cg3*	3.13	4.080 (2)	161
C37^iv^—H37*A* ^iv^⋯*Cg3*	3.36	4.260 (2)	153
C40^v^—H40*A* ^v^⋯C7	2.85	3.686 (2)	143

Symmetry codes: (i) −*x* + 1, *y*, −*z* + 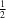; (ii) −*x* + 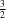, −*y* + 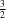, −*z*; (iv) −*x* + 1, −*y* + 2, −*z* + 1; (v) −*x* + 1, *y*, −*z* + 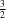.

**Table 3:** Experimental details

Crystal data	
Chemical formula	C_99_H_158_O_10_·C_2_H_4_Cl_2_
*M*_r_	1607.20
Crystal system, space group	Monoclinic, *C*2/*c*
Temperature (K)	150
*a*, *b*, *c* (Å)	31.4629 (12), 20.2692 (7), 15.3703 (11)
β (°)	91.275 (6)
*V* (Å^3^)	9799.7 (9)
*Z*	4
Radiation type	Mo *K*α
μ (mm^−1^)	0.12
Crystal size (mm)	0.21 × 0.13 × 0.09

Data collection	
Diffractometer	Rigaku R-AXIS RAPID
Absorption correction	Multi-scan (*ABSCOR*; Higashi, 1995 ▸)
*T*_min_, *T*_max_	0.774, 0.989
No. of measured, independent and observed [*I* > 2σ(*I*)] reflections	42520, 9959, 7023
*R*_int_	0.032
(sin θ/λ)_max_ (Å^−1^)	0.624

Refinement	
*R*[*F* ^2^ > 2σ(*F* ^2^)], *wR*(*F* ^2^), *S*	0.049, 0.137, 1.08
No. of reflections	9959
No. of parameters	528
No. of restraints	24
H-atom treatment	H atoms treated by a mixture of independent and constrained refinement
Δρ_max_, Δρ_min_ (e Å^−3^)	0.39, −0.43

Computer programs: *CrystalClear-SM Expert* (Rigaku, 2009 ▸), *CrystalStructure* (Rigaku, 2010 ▸), *Il Milione* (Burla *et al.*, 2007 ▸), *SHELXL2017/1* (Sheldrick, 2015 ▸), *ShelXle* (Hübschle *et al.*, 2011 ▸) and *Mercury* (Macrae *et al.*, 2006 ▸).

## supplementary crystallographic information

### Crystal data

**Table:**

C_99_H_158_O_10_·C_2_H_4_Cl_2_	*F*(000) = 3528
*M**_r_* = 1607.20	*D*_x_ = 1.089 Mg m^−^^3^
Monoclinic, *C*2/*c*	Mo *K*α radiation, λ = 0.71075 Å
*a* = 31.4629 (12) Å	Cell parameters from 11636 reflections
*b* = 20.2692 (7) Å	θ = 3.1–26.3°
*c* = 15.3703 (11) Å	µ = 0.12 mm^−^^1^
β = 91.275 (6)°	*T* = 150 K
*V* = 9799.7 (9) Å^3^	Block, colorless
*Z* = 4	0.21 × 0.13 × 0.09 mm

### Data collection

**Table:**

Rigaku R-AXIS RAPID diffractometer	7023 reflections with *I* > 2σ(*I*)
Detector resolution: 10.000 pixels mm^-1^	*R*_int_ = 0.032
ω scans	θ_max_ = 26.3°, θ_min_ = 3.1°
Absorption correction: multi-scan (ABSCOR; Higashi, 1995)	*h* = −37→39
*T*_min_ = 0.774, *T*_max_ = 0.989	*k* = −25→24
42520 measured reflections	*l* = −19→19
9959 independent reflections	

### Refinement

**Table:**

Refinement on *F*^2^	24 restraints
Least-squares matrix: full	Hydrogen site location: mixed
*R*[*F*^2^ > 2σ(*F*^2^)] = 0.049	H atoms treated by a mixture of independent and constrained refinement
*wR*(*F*^2^) = 0.137	*w* = 1/[σ^2^(*F*_o_^2^) + (0.064*P*)^2^ + 2.8339*P*] where *P* = (*F*_o_^2^ + 2*F*_c_^2^)/3
*S* = 1.08	(Δ/σ)_max_ = 0.001
9959 reflections	Δρ_max_ = 0.39 e Å^−^^3^
528 parameters	Δρ_min_ = −0.43 e Å^−^^3^

### Special details

**Table:**

Geometry. All esds (except the esd in the dihedral angle between two l.s. planes) are estimated using the full covariance matrix. The cell esds are taken into account individually in the estimation of esds in distances, angles and torsion angles; correlations between esds in cell parameters are only used when they are defined by crystal symmetry. An approximate (isotropic) treatment of cell esds is used for estimating esds involving l.s. planes.

### Fractional atomic coordinates and isotropic or equivalent isotropic displacement parameters (Å^2^)

**Table:**

	*x*	*y*	*z*	*U*_iso_*/*U*_eq_	Occ. (<1)
O1	0.52608 (3)	0.62986 (5)	0.52918 (7)	0.0395 (3)	
Cl1	0.55186 (2)	0.76990 (4)	0.16233 (5)	0.0952 (2)	
C51A	0.5073 (3)	0.7445 (4)	0.2239 (6)	0.125 (2)	0.464 (7)
H51A	0.484105	0.759512	0.184099	0.150*	0.464 (7)
H51B	0.508787	0.696374	0.213850	0.150*	0.464 (7)
C51B	0.51449 (18)	0.7915 (3)	0.2421 (5)	0.123 (2)	0.536 (7)
H51C	0.527534	0.769901	0.293884	0.148*	0.536 (7)
H51D	0.521354	0.838850	0.249897	0.148*	0.536 (7)
O2	0.64483 (3)	0.77669 (5)	0.35101 (7)	0.0433 (3)	
O3	0.51552 (3)	0.87675 (5)	0.51444 (7)	0.0392 (3)	
O4	0.58340 (3)	0.98033 (5)	0.21357 (7)	0.0407 (3)	
O5	0.47324 (4)	0.55783 (7)	0.42002 (8)	0.0535 (3)	
H5	0.4914 (7)	0.5732 (14)	0.4563 (15)	0.127 (11)*	
C1	0.57574 (4)	0.63729 (7)	0.41632 (10)	0.0342 (3)	
C2	0.55453 (4)	0.66856 (7)	0.48322 (10)	0.0340 (3)	
C3	0.56248 (4)	0.73431 (7)	0.50300 (10)	0.0344 (3)	
H3	0.547151	0.754950	0.548068	0.041*	
C4	0.59246 (4)	0.77054 (7)	0.45812 (10)	0.0331 (3)	
C5	0.61468 (4)	0.73893 (7)	0.39254 (10)	0.0353 (3)	
C6	0.60609 (4)	0.67346 (7)	0.37175 (10)	0.0360 (3)	
H6	0.621183	0.652917	0.326262	0.043*	
C7	0.59975 (5)	0.84281 (7)	0.47991 (10)	0.0363 (3)	
H7A	0.592112	0.850730	0.541169	0.044*	
H7B	0.630313	0.853176	0.474216	0.044*	
C8	0.57387 (4)	0.88849 (7)	0.42128 (10)	0.0330 (3)	
C9	0.53189 (4)	0.90490 (7)	0.44047 (10)	0.0334 (3)	
C10	0.50904 (5)	0.94760 (7)	0.38618 (10)	0.0335 (3)	
H10	0.480914	0.959667	0.400799	0.040*	
C11	0.52661 (4)	0.97304 (7)	0.31073 (10)	0.0324 (3)	
C12	0.56792 (4)	0.95468 (7)	0.29032 (10)	0.0329 (3)	
C13	0.59133 (4)	0.91376 (7)	0.34602 (10)	0.0344 (3)	
H13	0.619778	0.902890	0.332339	0.041*	
C14	0.500000	1.01589 (10)	0.250000	0.0336 (5)	
H14A	0.518806	1.044543	0.215644	0.040*	0.5
H14B	0.481194	1.044544	0.284356	0.040*	0.5
C15	0.48802 (5)	0.56267 (7)	0.33650 (10)	0.0363 (3)	
C16	0.53117 (5)	0.56392 (7)	0.31800 (10)	0.0348 (3)	
C17	0.54243 (5)	0.56370 (7)	0.23073 (10)	0.0372 (3)	
H17	0.571688	0.564275	0.216727	0.045*	
C18	0.56560 (5)	0.56684 (7)	0.38916 (10)	0.0378 (4)	
H18A	0.556069	0.541780	0.440451	0.045*	
H18B	0.591725	0.545572	0.367930	0.045*	
C19	0.49696 (5)	0.66342 (7)	0.58421 (10)	0.0384 (3)	
H19A	0.478500	0.693143	0.548950	0.046*	
H19B	0.512847	0.690397	0.627667	0.046*	
C20	0.47014 (5)	0.61268 (8)	0.62986 (11)	0.0410 (4)	
H20A	0.488771	0.583358	0.665336	0.049*	
H20B	0.454848	0.585243	0.586033	0.049*	
C21	0.43810 (5)	0.64623 (7)	0.68835 (10)	0.0387 (4)	
H21A	0.416887	0.669869	0.651436	0.046*	
H21B	0.453135	0.679399	0.724939	0.046*	
C22	0.41490 (5)	0.59824 (8)	0.74739 (11)	0.0410 (4)	
H22A	0.400150	0.564782	0.710783	0.049*	
H22B	0.436111	0.574949	0.784694	0.049*	
C23	0.38255 (5)	0.63133 (8)	0.80523 (10)	0.0408 (4)	
H23A	0.358628	0.647936	0.768461	0.049*	
H23B	0.396056	0.669711	0.834528	0.049*	
C24	0.36519 (5)	0.58494 (8)	0.87401 (11)	0.0440 (4)	
H24A	0.353325	0.545278	0.844775	0.053*	
H24B	0.388935	0.570446	0.912794	0.053*	
C25	0.33098 (6)	0.61590 (10)	0.92887 (12)	0.0547 (5)	
H25A	0.306286	0.627359	0.890791	0.066*	
H25B	0.342120	0.657311	0.954821	0.066*	
C26	0.31624 (6)	0.57082 (11)	1.00130 (12)	0.0643 (6)	
H26A	0.340499	0.559481	1.039500	0.077*	
H26B	0.304111	0.530447	0.975961	0.077*	
H26C	0.294599	0.593494	1.035103	0.077*	
C27	0.67085 (5)	0.74467 (9)	0.28883 (11)	0.0453 (4)	
H27A	0.652962	0.726534	0.240713	0.054*	
H27B	0.686720	0.707865	0.316701	0.054*	
C28	0.70150 (5)	0.79524 (9)	0.25407 (12)	0.0503 (4)	
H28A	0.717227	0.815997	0.303391	0.060*	
H28B	0.685263	0.830239	0.223085	0.060*	
C29	0.73314 (5)	0.76452 (10)	0.19214 (12)	0.0519 (5)	
H29A	0.750841	0.732111	0.224716	0.062*	
H29B	0.717211	0.740314	0.145944	0.062*	
C30	0.76207 (5)	0.81448 (10)	0.14993 (14)	0.0607 (5)	
H30A	0.776793	0.840235	0.196288	0.073*	
H30B	0.744354	0.845558	0.115134	0.073*	
C31	0.79542 (5)	0.78446 (11)	0.09109 (13)	0.0595 (5)	
H31A	0.815037	0.756882	0.126838	0.071*	
H31B	0.781035	0.755310	0.047959	0.071*	
C32	0.82113 (6)	0.83584 (11)	0.04310 (14)	0.0687 (6)	
H32A	0.834554	0.865875	0.086439	0.082*	
H32B	0.801450	0.862480	0.006255	0.082*	
C33	0.85581 (6)	0.80769 (12)	−0.01432 (13)	0.0695 (6)	
H33A	0.876784	0.783520	0.022559	0.083*	
H33B	0.842905	0.775904	−0.056065	0.083*	
C34	0.87843 (7)	0.86123 (14)	−0.06413 (18)	0.0942 (9)	
H34A	0.858974	0.879893	−0.108306	0.113*	
H34B	0.903232	0.842378	−0.092521	0.113*	
H34C	0.887728	0.896067	−0.023791	0.113*	
C35	0.47133 (5)	0.88783 (8)	0.52980 (10)	0.0373 (3)	
H35A	0.454098	0.873954	0.478320	0.045*	
H35B	0.466222	0.935390	0.539873	0.045*	
C36	0.45850 (5)	0.84864 (8)	0.60853 (10)	0.0409 (4)	
H36A	0.478914	0.857214	0.657167	0.049*	
H36B	0.459493	0.800958	0.594790	0.049*	
C37	0.41377 (5)	0.86709 (8)	0.63631 (11)	0.0420 (4)	
H37A	0.413354	0.914617	0.651120	0.050*	
H37B	0.393878	0.860306	0.586303	0.050*	
C38	0.39779 (5)	0.82805 (8)	0.71365 (11)	0.0427 (4)	
H38A	0.393628	0.781509	0.695872	0.051*	
H38B	0.419790	0.828902	0.760708	0.051*	
C39	0.35635 (5)	0.85437 (8)	0.74903 (11)	0.0444 (4)	
H39A	0.361483	0.899063	0.773012	0.053*	
H39B	0.335450	0.858617	0.700247	0.053*	
C40	0.33716 (5)	0.81172 (8)	0.81929 (10)	0.0399 (4)	
H40A	0.359749	0.800269	0.862559	0.048*	
H40B	0.326895	0.770091	0.792483	0.048*	
C41	0.30058 (5)	0.84390 (9)	0.86639 (12)	0.0497 (4)	
H41A	0.310896	0.885039	0.894424	0.060*	
H41B	0.278109	0.856038	0.823211	0.060*	
C42	0.28144 (6)	0.79983 (11)	0.93509 (13)	0.0605 (5)	
H42A	0.303696	0.786316	0.977014	0.073*	
H42B	0.269015	0.760640	0.907195	0.073*	
H42C	0.259266	0.824090	0.965415	0.073*	
C43	0.62086 (5)	0.95076 (8)	0.18009 (11)	0.0402 (4)	
H43A	0.617129	0.902359	0.175823	0.048*	
H43B	0.645483	0.959865	0.219482	0.048*	
C44	0.62861 (5)	0.97944 (8)	0.09101 (11)	0.0419 (4)	
H44A	0.631473	1.027953	0.095763	0.050*	
H44B	0.603891	0.969841	0.052082	0.050*	
C45	0.66860 (5)	0.95097 (9)	0.05176 (11)	0.0459 (4)	
H45A	0.664656	0.902882	0.043722	0.055*	
H45B	0.692667	0.957418	0.093479	0.055*	
C46	0.68018 (5)	0.98138 (9)	−0.03522 (11)	0.0445 (4)	
H46A	0.656030	0.975589	−0.076911	0.053*	
H46B	0.684781	1.029331	−0.027189	0.053*	
C47	0.71981 (5)	0.95118 (9)	−0.07369 (11)	0.0445 (4)	
H47A	0.714639	0.903585	−0.083778	0.053*	
H47B	0.743532	0.955125	−0.030597	0.053*	
C48	0.73333 (5)	0.98252 (9)	−0.15878 (11)	0.0479 (4)	
H48A	0.709840	0.977884	−0.202276	0.058*	
H48B	0.738095	1.030254	−0.149018	0.058*	
C49	0.77328 (5)	0.95275 (10)	−0.19575 (12)	0.0526 (4)	
H49A	0.796653	0.956595	−0.151846	0.063*	
H49B	0.768337	0.905198	−0.206667	0.063*	
C50	0.78709 (7)	0.98497 (12)	−0.27952 (13)	0.0736 (6)	
H50A	0.764757	0.979396	−0.324380	0.088*	
H50B	0.813378	0.964245	−0.298910	0.088*	
H50C	0.792062	1.032112	−0.269421	0.088*	

### Atomic displacement parameters (Å^2^)

**Table:**

	*U*^11^	*U*^22^	*U*^33^	*U*^12^	*U*^13^	*U*^23^
O1	0.0483 (6)	0.0309 (6)	0.0401 (6)	−0.0049 (4)	0.0156 (5)	0.0032 (5)
Cl1	0.0912 (4)	0.0950 (5)	0.1005 (5)	0.0188 (3)	0.0283 (4)	0.0261 (4)
C51A	0.138 (5)	0.078 (4)	0.163 (6)	−0.007 (4)	0.076 (4)	−0.020 (4)
C51B	0.133 (5)	0.076 (4)	0.164 (5)	−0.035 (3)	0.068 (4)	−0.037 (4)
O2	0.0369 (6)	0.0438 (6)	0.0497 (7)	−0.0064 (4)	0.0110 (5)	0.0078 (5)
O3	0.0412 (6)	0.0429 (6)	0.0337 (6)	−0.0041 (4)	0.0065 (4)	0.0063 (5)
O4	0.0408 (6)	0.0406 (6)	0.0410 (6)	0.0016 (4)	0.0100 (5)	0.0117 (5)
O5	0.0490 (7)	0.0694 (9)	0.0427 (7)	−0.0088 (6)	0.0145 (6)	−0.0071 (6)
C1	0.0340 (7)	0.0327 (8)	0.0359 (8)	0.0015 (6)	0.0029 (6)	0.0063 (6)
C2	0.0352 (8)	0.0335 (8)	0.0335 (8)	−0.0037 (6)	0.0037 (6)	0.0079 (6)
C3	0.0372 (8)	0.0351 (8)	0.0309 (8)	−0.0017 (6)	0.0020 (6)	0.0049 (6)
C4	0.0327 (7)	0.0343 (8)	0.0321 (8)	−0.0029 (6)	−0.0050 (6)	0.0077 (6)
C5	0.0307 (7)	0.0389 (9)	0.0364 (9)	−0.0029 (6)	0.0012 (6)	0.0104 (7)
C6	0.0342 (7)	0.0385 (9)	0.0355 (9)	0.0019 (6)	0.0042 (6)	0.0053 (7)
C7	0.0369 (8)	0.0377 (8)	0.0341 (8)	−0.0090 (6)	−0.0030 (6)	0.0038 (7)
C8	0.0360 (8)	0.0297 (8)	0.0332 (8)	−0.0093 (6)	−0.0034 (6)	0.0009 (6)
C9	0.0409 (8)	0.0290 (8)	0.0303 (8)	−0.0088 (6)	0.0030 (6)	−0.0016 (6)
C10	0.0366 (7)	0.0289 (8)	0.0351 (8)	−0.0040 (6)	0.0038 (6)	−0.0032 (6)
C11	0.0388 (8)	0.0239 (7)	0.0344 (8)	−0.0052 (5)	0.0005 (6)	−0.0026 (6)
C12	0.0365 (8)	0.0293 (8)	0.0329 (8)	−0.0083 (6)	0.0031 (6)	0.0027 (6)
C13	0.0331 (7)	0.0325 (8)	0.0378 (9)	−0.0066 (6)	0.0008 (6)	0.0024 (6)
C14	0.0387 (11)	0.0260 (10)	0.0363 (12)	0.000	0.0047 (9)	0.000
C15	0.0422 (8)	0.0298 (8)	0.0374 (9)	−0.0028 (6)	0.0132 (6)	−0.0027 (6)
C16	0.0401 (8)	0.0230 (7)	0.0416 (9)	0.0008 (6)	0.0082 (6)	0.0019 (6)
C17	0.0349 (8)	0.0315 (8)	0.0456 (10)	0.0035 (6)	0.0114 (6)	0.0046 (7)
C18	0.0408 (8)	0.0315 (8)	0.0415 (9)	0.0028 (6)	0.0075 (7)	0.0046 (7)
C19	0.0472 (9)	0.0322 (8)	0.0363 (9)	−0.0031 (6)	0.0106 (7)	0.0005 (7)
C20	0.0501 (9)	0.0333 (8)	0.0402 (9)	−0.0041 (7)	0.0138 (7)	0.0022 (7)
C21	0.0466 (9)	0.0314 (8)	0.0385 (9)	−0.0034 (6)	0.0088 (7)	0.0015 (7)
C22	0.0463 (9)	0.0355 (9)	0.0418 (9)	−0.0043 (6)	0.0121 (7)	0.0004 (7)
C23	0.0440 (9)	0.0409 (9)	0.0380 (9)	−0.0034 (7)	0.0092 (7)	−0.0015 (7)
C24	0.0461 (9)	0.0485 (10)	0.0379 (9)	−0.0108 (7)	0.0096 (7)	−0.0024 (7)
C25	0.0503 (10)	0.0733 (13)	0.0409 (10)	−0.0048 (9)	0.0123 (8)	−0.0020 (9)
C26	0.0587 (11)	0.0906 (16)	0.0444 (11)	−0.0184 (10)	0.0169 (9)	−0.0040 (10)
C27	0.0373 (8)	0.0530 (10)	0.0460 (10)	−0.0031 (7)	0.0104 (7)	0.0058 (8)
C28	0.0367 (8)	0.0550 (11)	0.0595 (12)	−0.0036 (7)	0.0092 (7)	0.0170 (9)
C29	0.0385 (9)	0.0698 (13)	0.0477 (11)	−0.0090 (8)	0.0064 (7)	0.0096 (9)
C30	0.0396 (9)	0.0731 (13)	0.0700 (14)	0.0017 (8)	0.0155 (9)	0.0266 (11)
C31	0.0412 (9)	0.0900 (15)	0.0475 (11)	−0.0105 (9)	0.0049 (8)	0.0104 (10)
C32	0.0406 (10)	0.0929 (16)	0.0732 (14)	0.0043 (9)	0.0163 (9)	0.0312 (12)
C33	0.0476 (11)	0.1114 (18)	0.0497 (12)	−0.0160 (11)	0.0077 (9)	0.0067 (12)
C34	0.0600 (13)	0.125 (2)	0.099 (2)	0.0011 (13)	0.0372 (13)	0.0313 (17)
C35	0.0415 (8)	0.0339 (8)	0.0366 (9)	−0.0028 (6)	0.0074 (6)	−0.0028 (7)
C36	0.0473 (9)	0.0374 (9)	0.0385 (9)	−0.0047 (7)	0.0096 (7)	0.0006 (7)
C37	0.0486 (9)	0.0350 (9)	0.0428 (10)	−0.0019 (7)	0.0111 (7)	−0.0003 (7)
C38	0.0445 (9)	0.0442 (10)	0.0397 (9)	−0.0008 (7)	0.0079 (7)	0.0026 (7)
C39	0.0488 (9)	0.0417 (9)	0.0432 (10)	−0.0013 (7)	0.0111 (7)	−0.0010 (7)
C40	0.0376 (8)	0.0474 (9)	0.0350 (9)	−0.0037 (7)	0.0020 (6)	−0.0005 (7)
C41	0.0451 (9)	0.0561 (11)	0.0484 (11)	−0.0038 (8)	0.0107 (7)	−0.0011 (8)
C42	0.0487 (10)	0.0798 (14)	0.0538 (12)	−0.0054 (9)	0.0149 (8)	0.0059 (10)
C43	0.0346 (8)	0.0426 (9)	0.0436 (10)	−0.0011 (6)	0.0062 (6)	0.0086 (7)
C44	0.0400 (8)	0.0434 (9)	0.0427 (10)	−0.0030 (7)	0.0065 (7)	0.0094 (7)
C45	0.0396 (8)	0.0524 (10)	0.0458 (10)	−0.0008 (7)	0.0071 (7)	0.0111 (8)
C46	0.0416 (9)	0.0476 (10)	0.0446 (10)	−0.0028 (7)	0.0071 (7)	0.0076 (8)
C47	0.0386 (8)	0.0490 (10)	0.0462 (10)	−0.0042 (7)	0.0065 (7)	0.0041 (8)
C48	0.0458 (9)	0.0543 (11)	0.0441 (10)	−0.0056 (7)	0.0076 (7)	0.0013 (8)
C49	0.0509 (10)	0.0589 (11)	0.0484 (11)	−0.0075 (8)	0.0107 (8)	−0.0092 (9)
C50	0.0720 (13)	0.1006 (18)	0.0492 (12)	−0.0119 (12)	0.0220 (10)	−0.0087 (12)

### Geometric parameters (Å, º)

**Table:**

O1—C2	1.3942 (16)	C27—C28	1.513 (2)
O1—C19	1.4323 (17)	C27—H27A	0.9900
Cl1—C51B	1.772 (5)	C27—H27B	0.9900
Cl1—C51A	1.785 (5)	C28—C29	1.525 (2)
C51A—C51A^i^	0.932 (11)	C28—H28A	0.9900
C51A—H51A	0.9900	C28—H28B	0.9900
C51A—H51B	0.9900	C29—C30	1.517 (2)
C51B—C51B^i^	0.949 (9)	C29—H29A	0.9900
C51B—H51C	0.9900	C29—H29B	0.9900
C51B—H51D	0.9900	C30—C31	1.527 (3)
O2—C5	1.3857 (17)	C30—H30A	0.9900
O2—C27	1.4280 (19)	C30—H30B	0.9900
O3—C9	1.3817 (17)	C31—C32	1.520 (2)
O3—C35	1.4332 (17)	C31—H31A	0.9900
O4—C12	1.3873 (17)	C31—H31B	0.9900
O4—C43	1.4282 (18)	C32—C33	1.529 (3)
O5—C15	1.3786 (19)	C32—H32A	0.9900
O5—H5	0.847 (10)	C32—H32B	0.9900
C1—C2	1.391 (2)	C33—C34	1.515 (3)
C1—C6	1.3955 (19)	C33—H33A	0.9900
C1—C18	1.520 (2)	C33—H33B	0.9900
C2—C3	1.388 (2)	C34—H34A	0.9800
C3—C4	1.3905 (19)	C34—H34B	0.9800
C3—H3	0.9500	C34—H34C	0.9800
C4—C5	1.395 (2)	C35—C36	1.510 (2)
C4—C7	1.519 (2)	C35—H35A	0.9900
C5—C6	1.390 (2)	C35—H35B	0.9900
C6—H6	0.9500	C36—C37	1.526 (2)
C7—C8	1.517 (2)	C36—H36A	0.9900
C7—H7A	0.9900	C36—H36B	0.9900
C7—H7B	0.9900	C37—C38	1.523 (2)
C8—C13	1.389 (2)	C37—H37A	0.9900
C8—C9	1.400 (2)	C37—H37B	0.9900
C9—C10	1.391 (2)	C38—C39	1.521 (2)
C10—C11	1.395 (2)	C38—H38A	0.9900
C10—H10	0.9500	C38—H38B	0.9900
C11—C12	1.395 (2)	C39—C40	1.519 (2)
C11—C14	1.5139 (18)	C39—H39A	0.9900
C12—C13	1.391 (2)	C39—H39B	0.9900
C13—H13	0.9500	C40—C41	1.520 (2)
C14—H14A	0.9900	C40—H40A	0.9900
C14—H14B	0.9900	C40—H40B	0.9900
C15—C16	1.394 (2)	C41—C42	1.518 (2)
C15—C17^i^	1.394 (2)	C41—H41A	0.9900
C16—C17	1.395 (2)	C41—H41B	0.9900
C16—C18	1.523 (2)	C42—H42A	0.9800
C17—H17	0.9500	C42—H42B	0.9800
C18—H18A	0.9900	C42—H42C	0.9800
C18—H18B	0.9900	C43—C44	1.512 (2)
C19—C20	1.5128 (19)	C43—H43A	0.9900
C19—H19A	0.9900	C43—H43B	0.9900
C19—H19B	0.9900	C44—C45	1.521 (2)
C20—C21	1.526 (2)	C44—H44A	0.9900
C20—H20A	0.9900	C44—H44B	0.9900
C20—H20B	0.9900	C45—C46	1.524 (2)
C21—C22	1.527 (2)	C45—H45A	0.9900
C21—H21A	0.9900	C45—H45B	0.9900
C21—H21B	0.9900	C46—C47	1.520 (2)
C22—C23	1.522 (2)	C46—H46A	0.9900
C22—H22A	0.9900	C46—H46B	0.9900
C22—H22B	0.9900	C47—C48	1.523 (2)
C23—C24	1.525 (2)	C47—H47A	0.9900
C23—H23A	0.9900	C47—H47B	0.9900
C23—H23B	0.9900	C48—C49	1.516 (2)
C24—C25	1.518 (2)	C48—H48A	0.9900
C24—H24A	0.9900	C48—H48B	0.9900
C24—H24B	0.9900	C49—C50	1.516 (3)
C25—C26	1.521 (3)	C49—H49A	0.9900
C25—H25A	0.9900	C49—H49B	0.9900
C25—H25B	0.9900	C50—H50A	0.9800
C26—H26A	0.9800	C50—H50B	0.9800
C26—H26B	0.9800	C50—H50C	0.9800
C26—H26C	0.9800		
			
C2—O1—C19	117.27 (11)	C29—C28—H28A	109.2
C51A^i^—C51A—Cl1	149.8 (14)	C27—C28—H28B	109.2
C51A^i^—C51A—H51A	99.2	C29—C28—H28B	109.2
Cl1—C51A—H51A	99.2	H28A—C28—H28B	107.9
C51A^i^—C51A—H51B	99.2	C30—C29—C28	113.61 (16)
Cl1—C51A—H51B	99.2	C30—C29—H29A	108.8
H51A—C51A—H51B	104.0	C28—C29—H29A	108.8
C51B^i^—C51B—Cl1	146.5 (11)	C30—C29—H29B	108.8
C51B^i^—C51B—H51C	100.2	C28—C29—H29B	108.8
Cl1—C51B—H51C	100.2	H29A—C29—H29B	107.7
C51B^i^—C51B—H51D	100.2	C29—C30—C31	114.46 (17)
Cl1—C51B—H51D	100.2	C29—C30—H30A	108.6
H51C—C51B—H51D	104.2	C31—C30—H30A	108.6
C5—O2—C27	117.77 (12)	C29—C30—H30B	108.6
C9—O3—C35	116.85 (12)	C31—C30—H30B	108.6
C12—O4—C43	117.21 (11)	H30A—C30—H30B	107.6
C15—O5—H5	111 (2)	C32—C31—C30	113.23 (18)
C2—C1—C6	117.92 (14)	C32—C31—H31A	108.9
C2—C1—C18	122.00 (13)	C30—C31—H31A	108.9
C6—C1—C18	120.04 (13)	C32—C31—H31B	108.9
C3—C2—C1	120.80 (13)	C30—C31—H31B	108.9
C3—C2—O1	122.95 (13)	H31A—C31—H31B	107.7
C1—C2—O1	116.24 (13)	C31—C32—C33	114.76 (19)
C2—C3—C4	121.29 (14)	C31—C32—H32A	108.6
C2—C3—H3	119.4	C33—C32—H32A	108.6
C4—C3—H3	119.4	C31—C32—H32B	108.6
C3—C4—C5	118.17 (13)	C33—C32—H32B	108.6
C3—C4—C7	120.05 (14)	H32A—C32—H32B	107.6
C5—C4—C7	121.77 (13)	C34—C33—C32	111.9 (2)
O2—C5—C6	123.57 (14)	C34—C33—H33A	109.2
O2—C5—C4	116.00 (13)	C32—C33—H33A	109.2
C6—C5—C4	120.43 (13)	C34—C33—H33B	109.2
C5—C6—C1	121.35 (14)	C32—C33—H33B	109.2
C5—C6—H6	119.3	H33A—C33—H33B	107.9
C1—C6—H6	119.3	C33—C34—H34A	109.5
C8—C7—C4	112.41 (12)	C33—C34—H34B	109.5
C8—C7—H7A	109.1	H34A—C34—H34B	109.5
C4—C7—H7A	109.1	C33—C34—H34C	109.5
C8—C7—H7B	109.1	H34A—C34—H34C	109.5
C4—C7—H7B	109.1	H34B—C34—H34C	109.5
H7A—C7—H7B	107.9	O3—C35—C36	109.10 (13)
C13—C8—C9	118.69 (14)	O3—C35—H35A	109.9
C13—C8—C7	120.19 (13)	C36—C35—H35A	109.9
C9—C8—C7	121.10 (13)	O3—C35—H35B	109.9
O3—C9—C10	123.59 (13)	C36—C35—H35B	109.9
O3—C9—C8	116.49 (13)	H35A—C35—H35B	108.3
C10—C9—C8	119.92 (13)	C35—C36—C37	111.09 (13)
C9—C10—C11	121.32 (13)	C35—C36—H36A	109.4
C9—C10—H10	119.3	C37—C36—H36A	109.4
C11—C10—H10	119.3	C35—C36—H36B	109.4
C10—C11—C12	118.49 (13)	C37—C36—H36B	109.4
C10—C11—C14	120.06 (12)	H36A—C36—H36B	108.0
C12—C11—C14	121.32 (12)	C38—C37—C36	114.43 (14)
O4—C12—C13	123.76 (13)	C38—C37—H37A	108.7
O4—C12—C11	115.97 (13)	C36—C37—H37A	108.7
C13—C12—C11	120.26 (13)	C38—C37—H37B	108.7
C8—C13—C12	121.24 (13)	C36—C37—H37B	108.7
C8—C13—H13	119.4	H37A—C37—H37B	107.6
C12—C13—H13	119.4	C39—C38—C37	113.42 (14)
C11—C14—C11^i^	109.98 (16)	C39—C38—H38A	108.9
C11—C14—H14A	109.7	C37—C38—H38A	108.9
C11^i^—C14—H14A	109.7	C39—C38—H38B	108.9
C11—C14—H14B	109.7	C37—C38—H38B	108.9
C11^i^—C14—H14B	109.7	H38A—C38—H38B	107.7
H14A—C14—H14B	108.2	C40—C39—C38	114.34 (14)
O5—C15—C16	122.80 (15)	C40—C39—H39A	108.7
O5—C15—C17^i^	116.80 (13)	C38—C39—H39A	108.7
C16—C15—C17^i^	120.35 (14)	C40—C39—H39B	108.7
C15—C16—C17	117.74 (14)	C38—C39—H39B	108.7
C15—C16—C18	122.32 (14)	H39A—C39—H39B	107.6
C17—C16—C18	119.93 (13)	C39—C40—C41	114.30 (14)
C15^i^—C17—C16	121.87 (13)	C39—C40—H40A	108.7
C15^i^—C17—H17	119.1	C41—C40—H40A	108.7
C16—C17—H17	119.1	C39—C40—H40B	108.7
C1—C18—C16	112.06 (12)	C41—C40—H40B	108.7
C1—C18—H18A	109.2	H40A—C40—H40B	107.6
C16—C18—H18A	109.2	C42—C41—C40	113.27 (15)
C1—C18—H18B	109.2	C42—C41—H41A	108.9
C16—C18—H18B	109.2	C40—C41—H41A	108.9
H18A—C18—H18B	107.9	C42—C41—H41B	108.9
O1—C19—C20	108.77 (12)	C40—C41—H41B	108.9
O1—C19—H19A	109.9	H41A—C41—H41B	107.7
C20—C19—H19A	109.9	C41—C42—H42A	109.5
O1—C19—H19B	109.9	C41—C42—H42B	109.5
C20—C19—H19B	109.9	H42A—C42—H42B	109.5
H19A—C19—H19B	108.3	C41—C42—H42C	109.5
C19—C20—C21	110.68 (12)	H42A—C42—H42C	109.5
C19—C20—H20A	109.5	H42B—C42—H42C	109.5
C21—C20—H20A	109.5	O4—C43—C44	108.42 (12)
C19—C20—H20B	109.5	O4—C43—H43A	110.0
C21—C20—H20B	109.5	C44—C43—H43A	110.0
H20A—C20—H20B	108.1	O4—C43—H43B	110.0
C20—C21—C22	113.40 (12)	C44—C43—H43B	110.0
C20—C21—H21A	108.9	H43A—C43—H43B	108.4
C22—C21—H21A	108.9	C43—C44—C45	111.40 (13)
C20—C21—H21B	108.9	C43—C44—H44A	109.3
C22—C21—H21B	108.9	C45—C44—H44A	109.3
H21A—C21—H21B	107.7	C43—C44—H44B	109.3
C23—C22—C21	113.65 (13)	C45—C44—H44B	109.3
C23—C22—H22A	108.8	H44A—C44—H44B	108.0
C21—C22—H22A	108.8	C44—C45—C46	114.25 (14)
C23—C22—H22B	108.8	C44—C45—H45A	108.7
C21—C22—H22B	108.8	C46—C45—H45A	108.7
H22A—C22—H22B	107.7	C44—C45—H45B	108.7
C22—C23—C24	112.81 (13)	C46—C45—H45B	108.7
C22—C23—H23A	109.0	H45A—C45—H45B	107.6
C24—C23—H23A	109.0	C47—C46—C45	113.11 (14)
C22—C23—H23B	109.0	C47—C46—H46A	109.0
C24—C23—H23B	109.0	C45—C46—H46A	109.0
H23A—C23—H23B	107.8	C47—C46—H46B	109.0
C25—C24—C23	113.55 (15)	C45—C46—H46B	109.0
C25—C24—H24A	108.9	H46A—C46—H46B	107.8
C23—C24—H24A	108.9	C46—C47—C48	114.40 (14)
C25—C24—H24B	108.9	C46—C47—H47A	108.7
C23—C24—H24B	108.9	C48—C47—H47A	108.7
H24A—C24—H24B	107.7	C46—C47—H47B	108.7
C24—C25—C26	112.95 (17)	C48—C47—H47B	108.7
C24—C25—H25A	109.0	H47A—C47—H47B	107.6
C26—C25—H25A	109.0	C49—C48—C47	113.93 (15)
C24—C25—H25B	109.0	C49—C48—H48A	108.8
C26—C25—H25B	109.0	C47—C48—H48A	108.8
H25A—C25—H25B	107.8	C49—C48—H48B	108.8
C25—C26—H26A	109.5	C47—C48—H48B	108.8
C25—C26—H26B	109.5	H48A—C48—H48B	107.7
H26A—C26—H26B	109.5	C50—C49—C48	113.74 (17)
C25—C26—H26C	109.5	C50—C49—H49A	108.8
H26A—C26—H26C	109.5	C48—C49—H49A	108.8
H26B—C26—H26C	109.5	C50—C49—H49B	108.8
O2—C27—C28	107.93 (14)	C48—C49—H49B	108.8
O2—C27—H27A	110.1	H49A—C49—H49B	107.7
C28—C27—H27A	110.1	C49—C50—H50A	109.5
O2—C27—H27B	110.1	C49—C50—H50B	109.5
C28—C27—H27B	110.1	H50A—C50—H50B	109.5
H27A—C27—H27B	108.4	C49—C50—H50C	109.5
C27—C28—C29	111.95 (15)	H50A—C50—H50C	109.5
C27—C28—H28A	109.2	H50B—C50—H50C	109.5
			
C6—C1—C2—C3	1.9 (2)	O4—C12—C13—C8	−178.75 (13)
C18—C1—C2—C3	−175.80 (14)	C11—C12—C13—C8	2.2 (2)
C6—C1—C2—O1	−176.77 (12)	C10—C11—C14—C11^i^	−83.32 (12)
C18—C1—C2—O1	5.5 (2)	C12—C11—C14—C11^i^	92.57 (13)
C19—O1—C2—C3	15.2 (2)	O5—C15—C16—C17	175.06 (13)
C19—O1—C2—C1	−166.12 (13)	C17^i^—C15—C16—C17	−2.36 (19)
C1—C2—C3—C4	−1.4 (2)	O5—C15—C16—C18	−6.2 (2)
O1—C2—C3—C4	177.16 (13)	C17^i^—C15—C16—C18	176.41 (13)
C2—C3—C4—C5	−0.3 (2)	C15—C16—C17—C15^i^	0.37 (19)
C2—C3—C4—C7	178.65 (13)	C18—C16—C17—C15^i^	−178.43 (13)
C27—O2—C5—C6	−5.6 (2)	C2—C1—C18—C16	90.31 (17)
C27—O2—C5—C4	174.64 (13)	C6—C1—C18—C16	−87.37 (16)
C3—C4—C5—O2	−178.67 (12)	C15—C16—C18—C1	−87.07 (17)
C7—C4—C5—O2	2.4 (2)	C17—C16—C18—C1	91.68 (16)
C3—C4—C5—C6	1.5 (2)	C2—O1—C19—C20	−178.21 (12)
C7—C4—C5—C6	−177.41 (13)	O1—C19—C20—C21	−179.15 (13)
O2—C5—C6—C1	179.18 (13)	C19—C20—C21—C22	−170.72 (14)
C4—C5—C6—C1	−1.0 (2)	C20—C21—C22—C23	−179.39 (14)
C2—C1—C6—C5	−0.7 (2)	C21—C22—C23—C24	−170.36 (14)
C18—C1—C6—C5	177.07 (13)	C22—C23—C24—C25	−176.61 (14)
C3—C4—C7—C8	−94.67 (16)	C23—C24—C25—C26	−175.88 (14)
C5—C4—C7—C8	84.26 (17)	C5—O2—C27—C28	−178.92 (13)
C4—C7—C8—C13	−92.92 (16)	O2—C27—C28—C29	175.51 (14)
C4—C7—C8—C9	85.65 (17)	C27—C28—C29—C30	175.15 (15)
C35—O3—C9—C10	6.3 (2)	C28—C29—C30—C31	177.17 (16)
C35—O3—C9—C8	−173.83 (12)	C29—C30—C31—C32	174.53 (16)
C13—C8—C9—O3	177.87 (12)	C30—C31—C32—C33	178.18 (17)
C7—C8—C9—O3	−0.7 (2)	C31—C32—C33—C34	176.70 (19)
C13—C8—C9—C10	−2.2 (2)	C9—O3—C35—C36	175.07 (12)
C7—C8—C9—C10	179.17 (13)	O3—C35—C36—C37	170.97 (12)
O3—C9—C10—C11	−177.94 (13)	C35—C36—C37—C38	178.06 (13)
C8—C9—C10—C11	2.2 (2)	C36—C37—C38—C39	170.93 (14)
C9—C10—C11—C12	0.1 (2)	C37—C38—C39—C40	173.32 (14)
C9—C10—C11—C14	176.09 (13)	C38—C39—C40—C41	169.14 (14)
C43—O4—C12—C13	15.6 (2)	C39—C40—C41—C42	178.94 (15)
C43—O4—C12—C11	−165.35 (12)	C12—O4—C43—C44	172.32 (12)
C10—C11—C12—O4	178.63 (12)	O4—C43—C44—C45	179.04 (13)
C14—C11—C12—O4	2.7 (2)	C43—C44—C45—C46	−175.75 (14)
C10—C11—C12—C13	−2.2 (2)	C44—C45—C46—C47	−178.99 (14)
C14—C11—C12—C13	−178.20 (13)	C45—C46—C47—C48	−177.51 (14)
C9—C8—C13—C12	0.1 (2)	C46—C47—C48—C49	179.06 (14)
C7—C8—C13—C12	178.69 (13)	C47—C48—C49—C50	−178.88 (15)

Symmetry code: (i) −*x*+1, *y*, −*z*+1/2.

### Hydrogen-bond geometry (Å, º)

**Table:**

*D*—H···*A*	*D*—H	H···*A*	*D*···*A*	*D*—H···*A*
O5—H5···O1	0.85 (2)	1.93 (2)	2.754 (2)	165 (2)
C34^ii^—H34*B*^ii^···Cl1	0.98	2.90	3.782 (3)	151

Symmetry code: (ii) −*x*+3/2, −*y*+3/2, −*z*.
